# Supplementary material for: Structural barriers control the spatial extent of slow earthquake slip
Source: Nat Commun. 2026 Jan 3;17:1431. doi: 10.1038/s41467-025-68179-1 (PMC12881577; doi:10.1038/s41467-025-68179-1)
Supplement: Supplementary file 1 — Supplementary Information [file 41467_2025_68179_MOESM1_ESM.pdf]

Supplementary Information for

Structural barriers control the spatial extent of slow earthquake slip

Takeshi Akuhara[1*], Kazuya Shiraishi[2], Takeshi Tsuji[3], Yusuke Yamashita[4], Hiroko Sugioka[5], Atikul Haque Farazi[6], Shukei Ohyanagi[7], Yoshihiro Ito[8], Ryuta Arai[2], Eiichiro Araki[2], Gou Fujie[2], Yasuyuki Nakamura[2], Takashi Tonegawa[2], Ryosuke Azuma[9], Ryota Hino[9], Kimihiro Mochizuki[1], Shunsuke Takemura[1], Tomoaki Yamada[1], and Masanao Shinohara[1]

[1] Earthquake Research Institute, The University of Tokyo

[2] Research Institute for Marine Geodynamics, Japan Agency for Marine-Earth Science and Technology

[3] School of Engineering, The University of Tokyo

[4] Faculty of Humanities, Miyazaki Municipal University

[5] Graduate School of Science, Kobe University

[6] Department of Geology and Mining, University of Barishal

[7] Graduate school of science, Kyoto University

[8] Disaster Prevention Research Institute, Kyoto University

[9] Graduate School of Science, Tohoku University

*Corresponding author: Takeshi Akuhara (akuhara@eri.u-tokyo.ac.jp)

Contents of this file

Supplementary Figs. 1–11

Supplementary Table 1

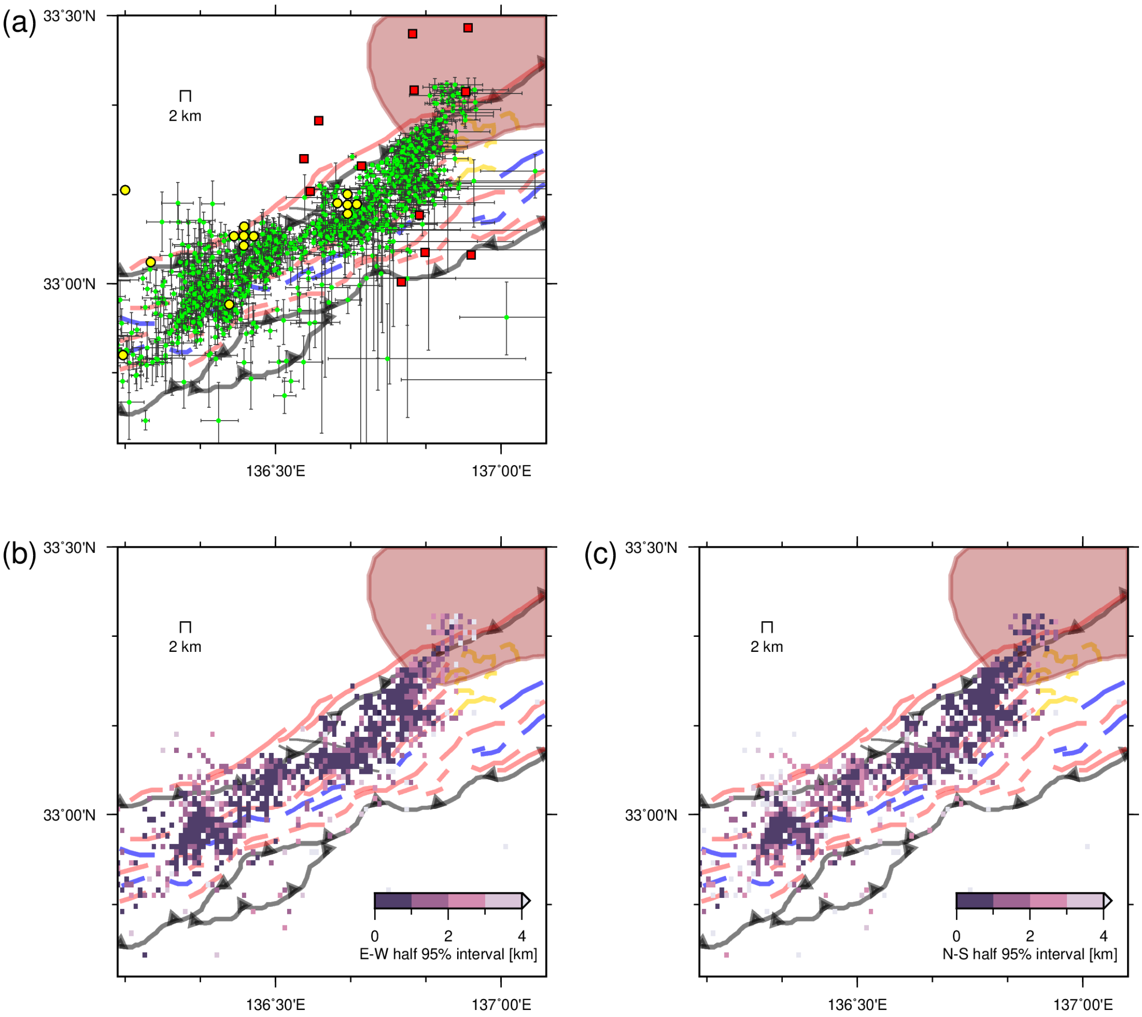

Supplementary Figure 1. Tremor locations and associated errors determined by inversion analysis of ref.[30]. (a) The green dots with error bars denote the event locations and associated uncertainties represented by the 95% confidence interval. The red squares and yellow circles represent the seismic stations (as shown in Fig. 1 of the main text). The background geological interpretation is the same as that shown in Fig. 2b of the main text. (b, c) The median of half the 95% confidence interval is shown for each 0.01° ×0.01° cell (approximately 1 km × 1 km), in the (b) east–west and (c) north–south directions. The position of the subducted Paleo–Zenisu ridge (red-shaded area) is based on ref.[40] and is reproduced with permission from Elsevier.

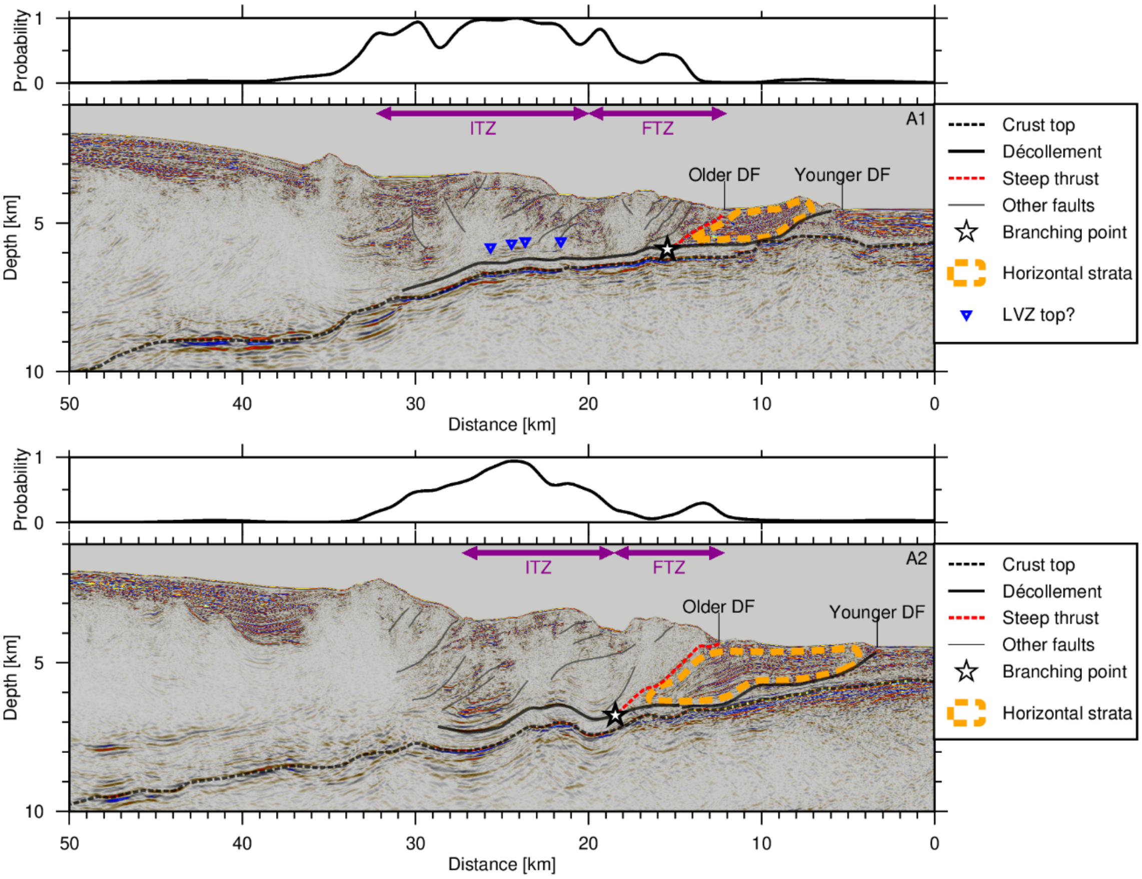

Supplementary Figure 2. Enlarged views of the seismic reflection profiles along survey lines A1 (top) and A2 (bottom). Notations are the same as those used in Fig. 3 of the main text. The dashed orange lines enclose the regions where horizontal strata are well-preserved. The blue inverted triangles indicate a reflector potentially indicating the top of a low-velocity zone.

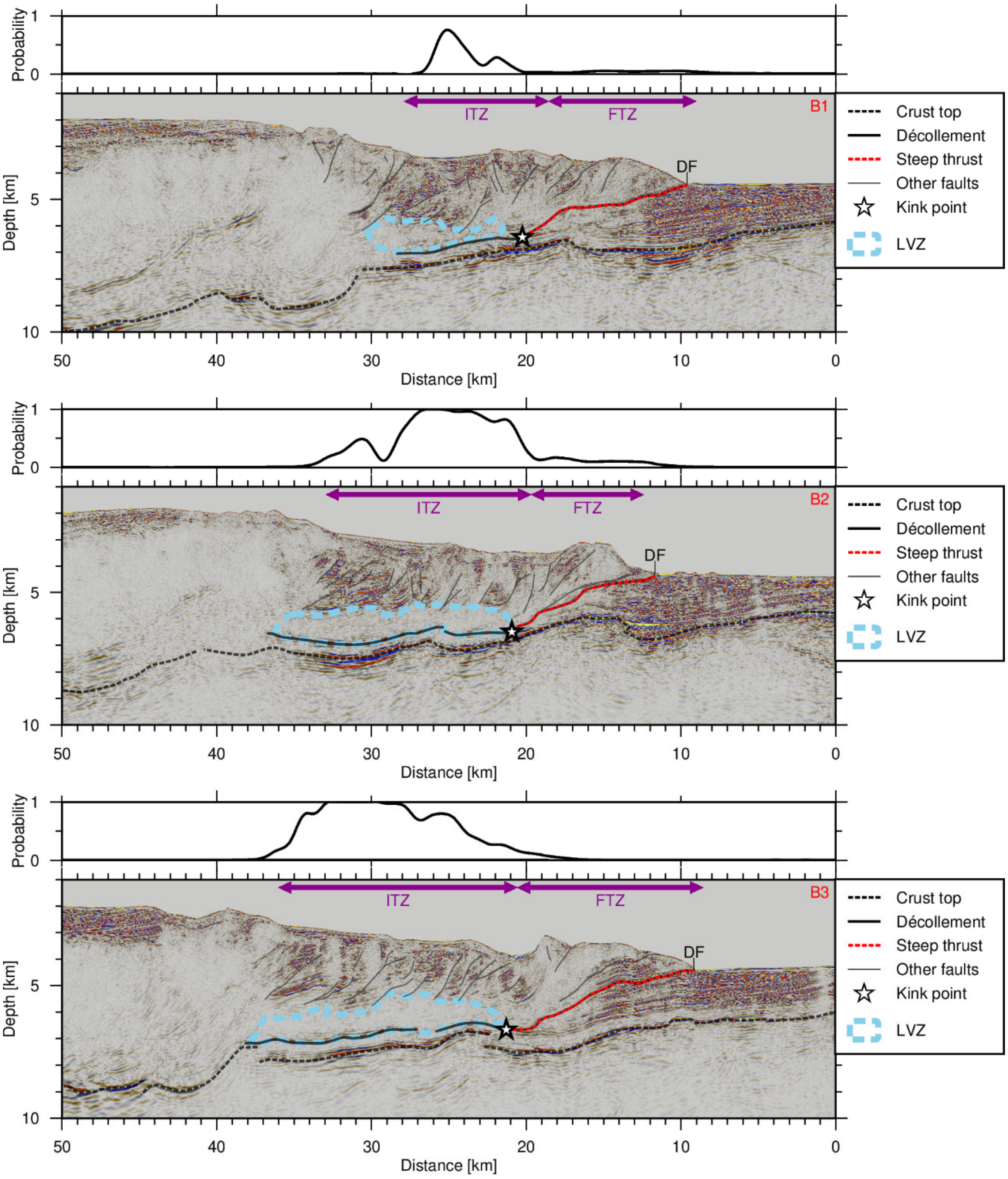

Supplementary Figure 3. Enlarged views of the seismic reflection profiles along survey lines B1 (top), B2 (middle), and B3 (bottom). Notations are the same as those used in Fig. 3 of the main text. The dashed blue lines enclose the regions of transparent seismic facies for which low seismic velocity is inferred.

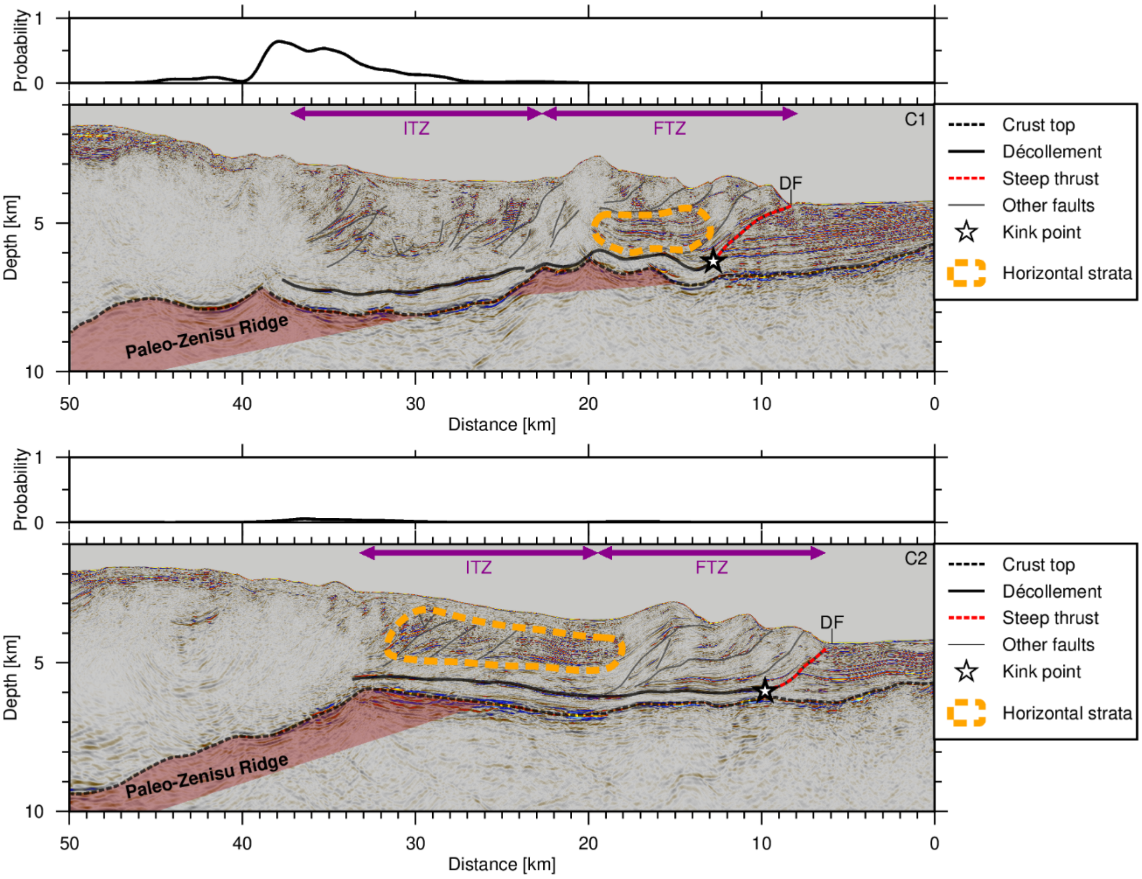

Supplementary Figure 4. Enlarged views of the seismic reflection profile along survey lines C1 (top) and C2 (bottom). Notations are the same as those used in Fig. 3 of the main text. The dashed orange lines enclose the regions where horizontal strata are well-preserved. The red shaded-areas show the subducted Paleo–Zenisu ridge and a smaller, undocumented ridge.

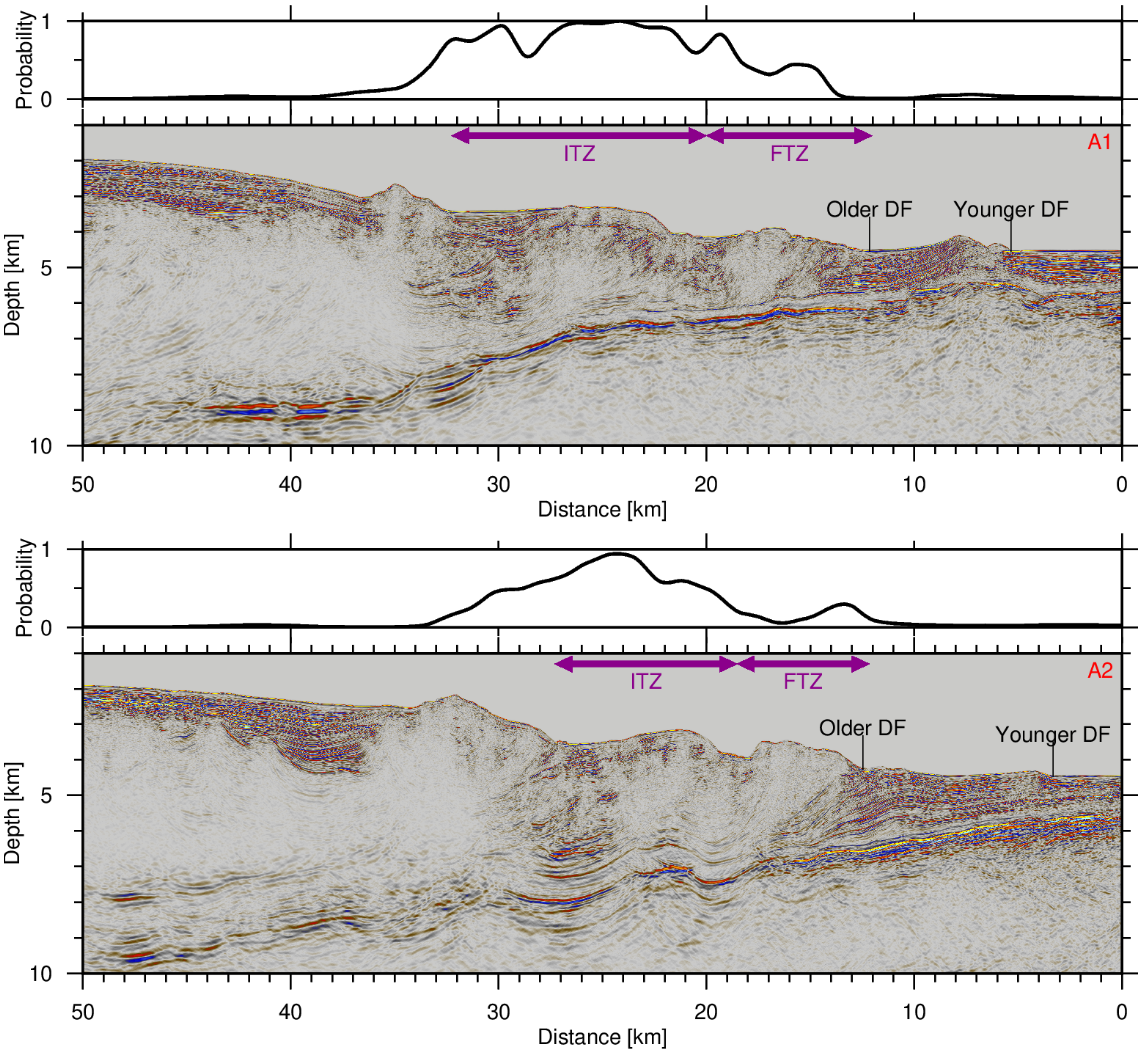

Supplementary Figure 5. Same as Supplementary Fig. 2, but with interpretations omitted.

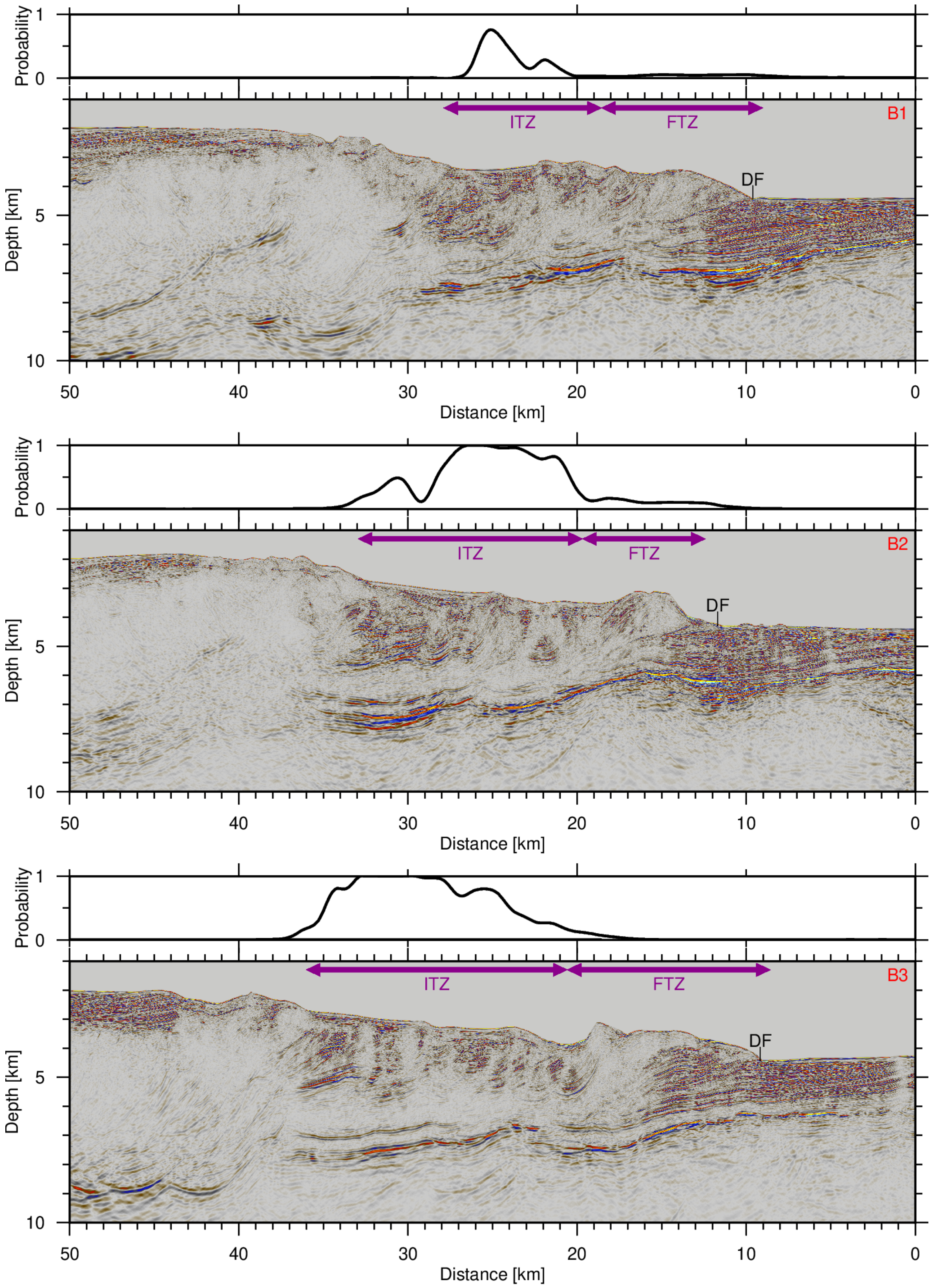

Supplementary Figure 6. Same as Supplementary Fig. 3, but with interpretations omitted.

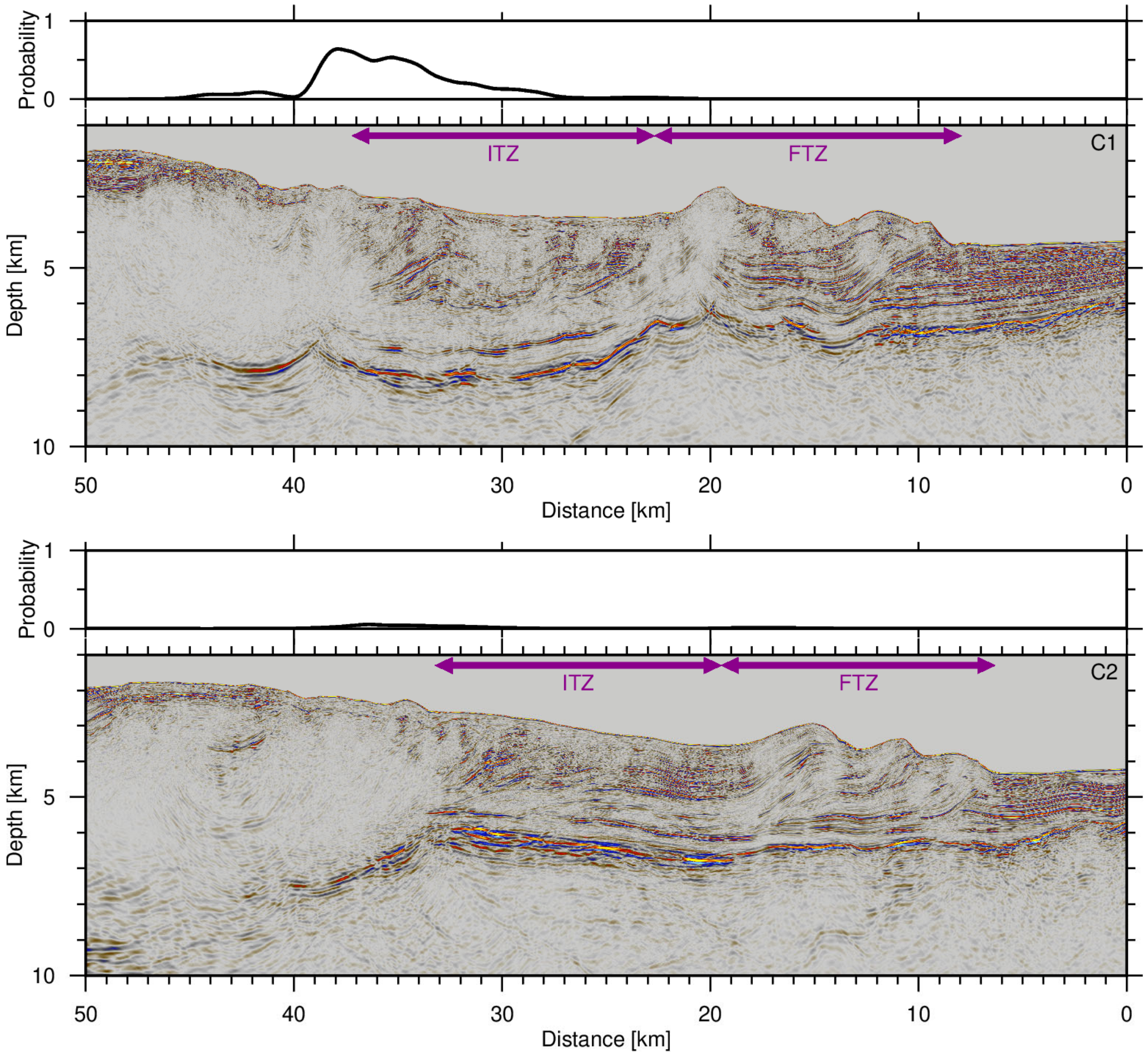

Supplementary Figure 7. Same as Supplementary Fig. 4, but with interpretations omitted.

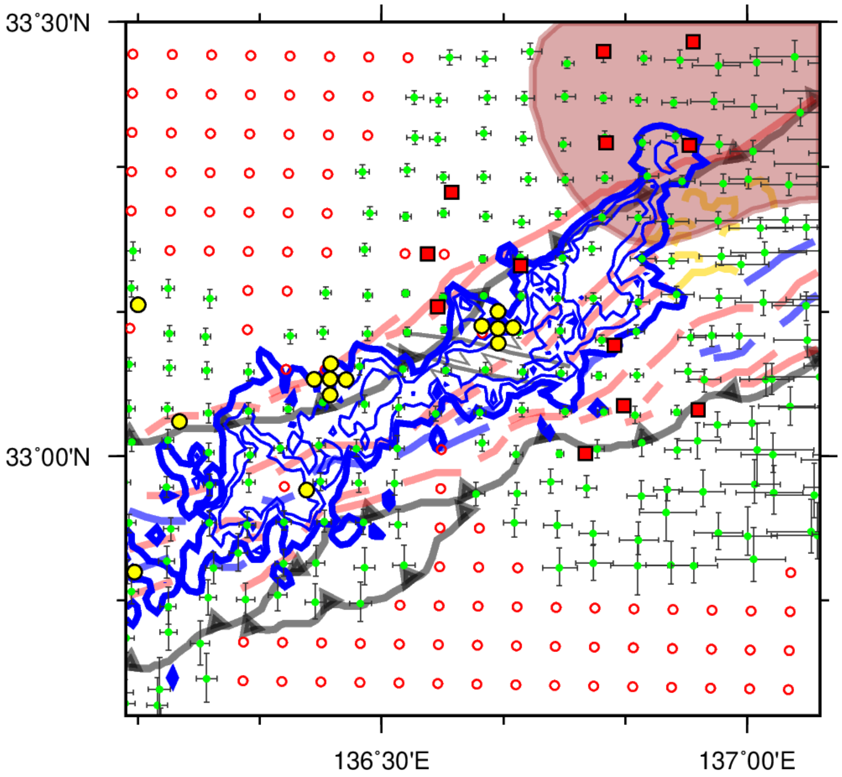

Supplementary Figure 8. Results of the synthetic test to check the reliability of the tremor locations. The green dots with error bars denote the estimated event locations and associated uncertainties represented by the 95% confidence interval. The red circles indicate the locations of events that were discarded during the prescreening. The blue contour lines represent the tremor probability with the same notation as that used in Fig. 2a of the main text. The other notations are the same as those used in Supplementary Fig. 1. The position of the subducted Paleo–Zenisu ridge (red-shaded area) is based on ref.[40] and is reproduced with permission from Elsevier.

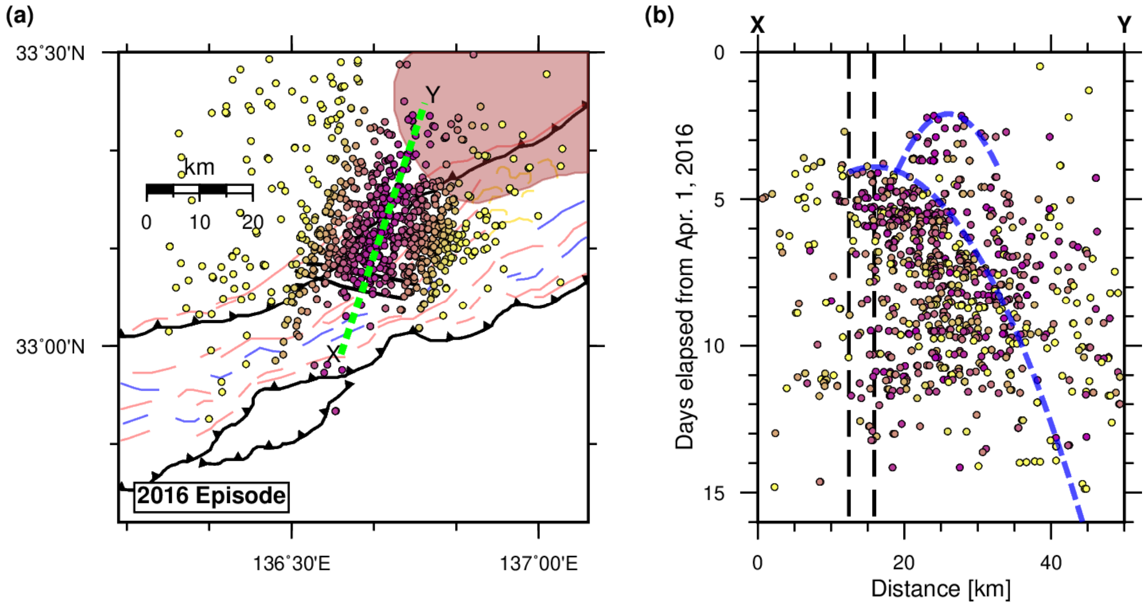

Supplementary Figure 9. Tremor locations during the 2016 episode, from April 1 to 15, 2016. (a) Circles denote the locations determined in a previous study[49], with their colors indicating the distance from the projection line (green dashed line). The background geological interpretation is the same as that shown in Fig. 2b of the main text. (b) Time–distance plot of tremors along the green dashed line shown in (a). The color notation is the same as in (a). The blue dashed parabola delineates the diffusive migrations of the tremors. The two vertical dashed lines represent the location of the strike-slip faults. The position of the subducted Paleo–Zenisu ridge (red-shaded area) is based on ref.[40] and is reproduced with permission from Elsevier.

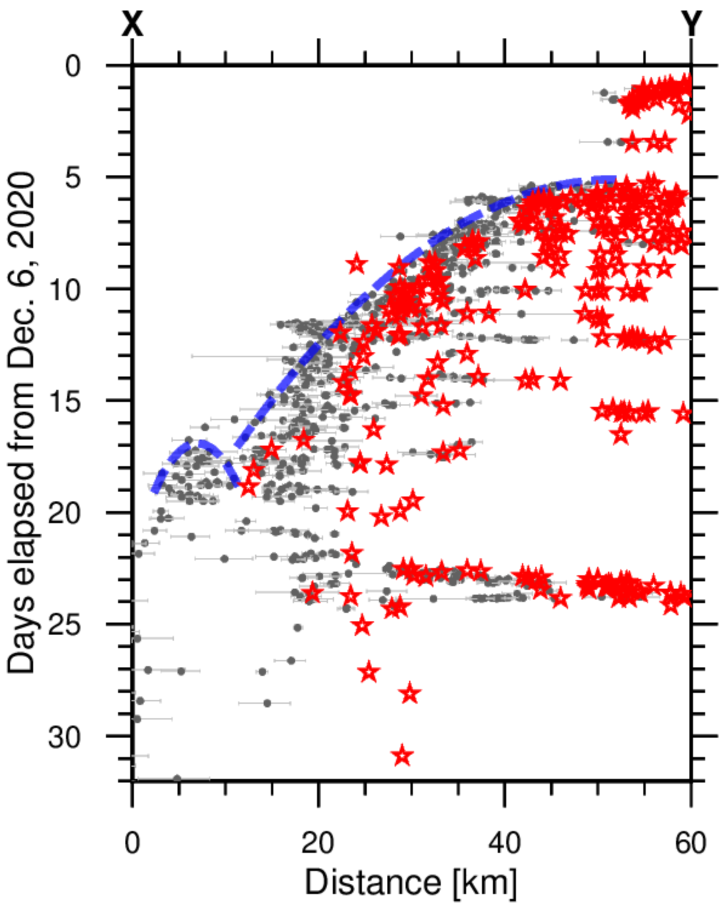

Supplementary Figure 10. Comparison of the spatiotemporal evolution of tremors and very low frequency earthquakes (VLFEs). Gray dots show the location of tremors along the XY profile in Fig. 5c of the main text, and red stars denote the location of VLFEs determined in a previous study[56]. The other notations follow those of Fig. 5c in the main text.

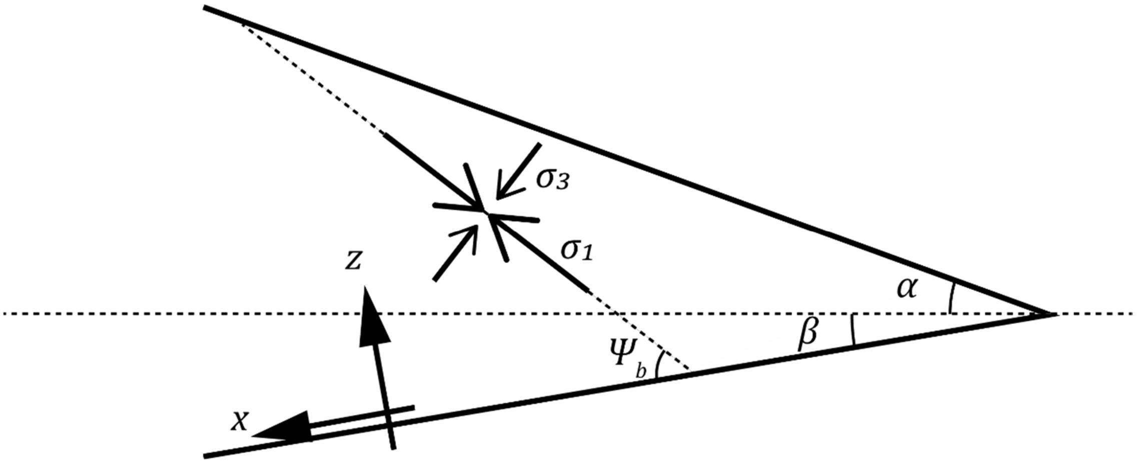

Supplementary Figure 11. Schematic illustration of the coordinate system and notation used in the calculation based on the Coulomb wedge theory.

Supplementary Table 1. Parameters assumed in the critical taper models and resulting stress orientation. All parameter values are adopted from ref.[42].

Tectonic domain	Surface slope angle, α (°)	Dip angle of the décollement, β (°)	Pore fluid pressure ratio, λ	Friction coefficient, μ_w	Orientation of the maximum principal stress axis, ψ_b (°)
ITZ	2.9	0.0	0.5	0.54	5.43
FTZ	10.0	7.6	0.5	0.57	26.2
